# Spiritual Holy Water Sites in Ethiopia: Unrecognized High-Risk Settings for Transmission of Pulmonary Tuberculosis

**DOI:** 10.1155/2024/3132498

**Published:** 2024-04-08

**Authors:** Melese Abate Reta, Nontuthuko Excellent Maningi, Gizachew Yismaw Wubetu, Steve A. S. Olorunju, P. Bernard Fourie

**Affiliations:** ^1^Research Centre for Tuberculosis and Department of Medical Microbiology, Faculty of Health Sciences, University of Pretoria, Prinshof, Pretoria 0084, South Africa; ^2^Department of Medical Laboratory Science, College of Health Sciences, Woldia University, P.O. Box 400, Woldia, Ethiopia; ^3^Department of Microbiology, School of Life Sciences, College of Agriculture, Engineering and Science, University of KwaZulu-Natal, Durban, South Africa; ^4^Amhara Public Health Institute, Bahir Dar, Ethiopia; ^5^Centre for Innovative Drug Development and Therapeutic Trials for Africa (CDT-Africa), College of Health Sciences, Addis Ababa University, Addis Ababa, Ethiopia; ^6^South African Medical Research Council, Biostatistics Unit, Pretoria, South Africa

## Abstract

Ethiopia is a high-tuberculosis (TB) burden country with 157 new cases per 100,000 people, with 23,800 TB-related deaths in 2020. In Ethiopia, TB patients have different healthcare-seeking behaviors. They frequently visit spiritual places, such as holy water sites (HWSs), to seek treatment for their illness spiritually. This study examined the prevalence of pulmonary TB (PTB) and drug susceptibility profiles of *Mycobacterium tuberculosis* (MTB) isolates among spiritual HWS attendees in Northwest Ethiopia. A cross-sectional study was conducted from June 2019 to March 2020. Sputum samples were collected, processed, and cultured using Löwenstein–Jensen (LJ) culture medium. Second-generation line probe assays (LPAs), GenoType®MTBDR*plus* VER2.0 and GenoType®MTBDR*sl* VER2.0, were used to detect anti-TB drug-resistant isolates. STATA 17 was utilized to perform descriptive statistics, bivariate, and multivariate regression analyses. Of 560 PTB-symptomatic participants, 21.8% ((95% confidence interval (95 CI): 18.4–25.2%)) were culture-positive, resulting in a point prevalence of 1,183/100,000 attendees. Amongst HWS attendees, culture-positive TB occurred most commonly in persons 18–33 years of age (28.5% (95 CI 23.4–34.3%)). Other participant characteristics significantly associated with culture-positive PTB were as follows: rural residents (adjusted odds ratio (aOR) 2.65; 95 CI 1.38–5.10), married participants (aOR 2.43; 95 CI 1.28–4.63), family members >5 per household (aOR 1.84; 95 CI 1.04–3.24), and sharing living space (aOR 10.57; 95 CI 3.60–31.13). Also, among 438 participants followed for 12 months after showing negative TB culture results while at the HWS, 6.8% (95 CI 4.4–9.4%) developed or contracted culture-positive TB post-residency at the HWSs. Of the 122 tested isolates, 20 (16.4%) were isoniazid (INH) and/or rifampicin (RIF) resistant. Multidrug-resistant (MDR) TB was detected in 15 cases (12.3%), five of which were fluoroquinolones (FLQs) resistant. The findings from this study should raise a concern about HWSs as potential high-risk settings for TB transmission. It is recommended that appropriate control measures be instituted that include compulsory TB testing and tightened infection control at HWSs, where an increased risk exists for transmission of TB.

## 1. Introduction

Tuberculosis (TB) remains to be a major global health issue [1, 2]. Worldwide, “10.0 million people were infected with TB and over 1.5 million died” from it in 2020 [2]. Until the 2020 coronavirus disease (COVID-19) pandemic, TB was the major infectious agent-related cause of mortality globally [2]. Although TB is a global issue, its prevalence, public health, and economic impact vary greatly between nations [1, 2]. TB is the leading cause of mortality among infectious diseases in low- and middle-income countries (LMICs) due to several factors. Poor healthcare access, overcrowded living conditions, poor nutrition, and the HIV/AIDS pandemic all contribute to the high burden of TB in LMICs [2–4]. Ethiopia, like other LMICs, has a high burden of TB, which poses challenges to its public healthcare system [2, 5, 6]. Ethiopia's TB incidence was 157 per 100,000 persons, with 23,800 people dying from the disease in 2020 [1], suggesting that TB continues a significant cause of death in the country.

The emergence of drug-resistant TB (DR-TB) has posed a significant threat to global and national TB control efforts, as DR-TB has the potential to spread globally, emphasizing the need for additional prevention and care measures [1, 2]. A recent national report revealed that the prevalence of MDR or RIF-resistant TB (MDR/RR-TB) in Ethiopia was 0.71% among newly diagnosed TB cases and 12.0% in retreated TB patients [1]. Besides, “pre-extensively drug-resistant (pre-XDR)” TB and “extensively drug-resistant (XDR)” TB were 5.7% and 0.6%, respectively [7]. Since the country lacks the facility to undertake universal drug susceptibility testing (DST) on all incident TB cases, early TB case detection and initiation of proper treatment are problematic in many public health facilities in Ethiopia [8].

Early TB case detection, successful TB patient treatment according to international standards, and TB prevention are global plans to halt TB and reduce deaths and transmission [2, 9]. However, “a key obstacle to achieving this goal has been that many people with TB are currently being” missed by healthcare systems [1]. Globally, over 2.9 million TB cases were anticipated to be underdiagnosed or diagnosed but unreported, most of which occurred in LMICs with weak healthcare systems [1]. Finding these cases, effectively diagnosing them, and commencing appropriate treatment are critical to containing the disease [1, 10]. In Ethiopia, about two-thirds of individuals with active TB in the community remain undiagnosed and thus untreated [11, 12]. This could be because the Ethiopian TB prevention and control program relies mostly on passive case finding, which requires TB suspects to self-present and visit public healthcare facilities [12]. However, studies showed that individuals with TB symptoms in LMICs, such as Ethiopia, exhibit different healthcare-seeking behaviors and use other methods before seeking treatment at public healthcare facilities [13–15]. Besides, people from poor communities have insufficient knowledge about TB disease, and they often confuse the symptoms of TB as indicative of other infections [14]. Moreover, healthcare facilities are not nearby to give TB services, and people with TB symptoms often live in places where government services have a hard time reaching, due to travel costs and fear of stigma, TB patients may not visit public healthcare facilities for diagnosis and treatment [14, 15]. Thus, TB patients can delay the opportunity for early diagnosis and the initiation of appropriate treatments. This can intensify and threaten TB transmission in households and the community.

In Ethiopia, people use spiritual holy water as an alternative treatment for a variety of diseases, including respiratory ailments [13, 16–18]. Holy water sites (HWSs) are designated areas with springs that are believed to have the power to cure various types of illness [13]. Although the number of people who use holy water as a treatment option is not well documented in the country, it is known that attendees believe in its healing power and use it as an alternative treatment for various illnesses. Particularly, Ethiopian Orthodox Tewahedo Christians have unwavering faith in the curative power of spiritual holy water [13, 16]. Thus, individuals from different parts of the country travel to HWSs to seek the curative power of holy water blessed by Orthodox priests and they reside in shared living spaces (rooms) for a certain period. The rooms are built as temporary waiting spaces, and they are small, overcrowded, and not well-ventilated, which can increase the risk of TB and other respiratory disease transmissions [13, 19].

In the Amhara region, the focus of this study, over 82.5% of the population are Ethiopian Orthodox Tewahedo Christian followers [13, 20], and faith-based therapy with spiritual holy water for TB and other diseases is widely practiced [13]. Although studies show that TB patients seek care from traditional healers and spiritual HWSs, the burden of TB and the drug resistance pattern of MTB isolates among HWS attendees in Ethiopia have not been thoroughly investigated. To the best of our knowledge, one earlier study has shown that “the prevalence of PTB among HWS attendees was 7.4-fold higher than the prevalence in the general population in Ethiopia” [13]. However, this study covered a limited geographical area and used smear microscopy to diagnose TB in symptomatic persons; given the low sensitivity of this technique, its findings might not reflect the true TB burden among this cohort of populations in the region. Hence, identifying such special congregate settings in the indigenous communities, considering these settings as hotspot sites for TB transmission, and conducting a systematic TB screening would aid in reaching those unreachable, missed, or undiagnosed TB cases for timely diagnosis and care. Therefore, this study aimed to assess the prevalence of TB and drug susceptibility profiles of MTB isolates among individuals with symptoms of PTB attending spiritual HWSs in the Amhara region, Ethiopia.

## 2. Methods

### 2.1. Study Setting

**Figure 1:**
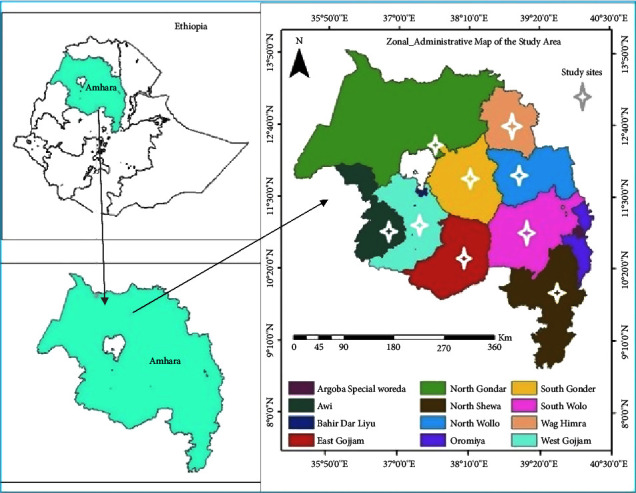
The study area map.

The Amhara region is located in the northwestern parts of Ethiopia and comprises eleven zones and three administrative towns (Figure 1). A cross-sectional study was done between June 2019 and March 2020 in nine purposefully selected HWSs found across nine administrative zones in the region. One HWS was chosen from each of the study zones. The HWS in each zone was chosen based on its consistent popularity for holy water treatment, its ability to accommodate many attendees, and where many people visit it throughout the year and stay for an extended time [13, 18] (Figure 1 and Table S1).

### 2.2. Population, Study Participants, and Recruitment

The study population comprised all attendees at the HWSs during the data collection period [18]. Following TB screening criteria, individuals with a persistent cough lasting two weeks or longer and other PTB-suggestive symptoms, such as productive cough or expectorating blood-containing sputum, fever, chest pain, shortness of breath, fatigue, night sweating, loss of appetite, unexplained weight loss, and contact history with active TB patients and history of TB disease, were screened [5]. A total of 10,313 attendees (≥18 years of age) were screened at nine selected HWSs during the study period; 560 of these individuals exhibited symptoms of PTB and participated. The study settings, total attendees screened for PTB-suggestive symptoms, anticipated PTB-symptomatic attendees from each study site, total participants, and laboratory test results are depicted in the additional files (Table S1).

### 2.3. Eligibility Criteria

Inclusion criteria: Individuals who were ≥18 years of age and fulfilled the screening criteria were included in the study [5, 21]. Exclusion criteria: Individuals who were seriously ill and unable to provide sputum samples and other relevant clinical or demographic information were excluded. Moreover, individuals who were receiving anti-TB treatment during data collection, and those whose permanent address was outside of the study region were excluded.

### 2.4. Screening and Socio-Demographic Data Collection

All attendees were screened for symptoms suggestive of PTB, following the guidelines [5, 21]. Trained nurses and medical laboratory technologists with experience in TB screening and similar field data collection perform the data collection. Descriptive demographic data were collected from eligible HWS attendees by interviewer-administered questionnaire. Participants' socio-demographic data included their sex, age, marital status, educational status, household size, and place of residence, as well as risk factors for PTB infection such as a history of TB disease, contact with active TB patients or person with a chronic cough, family history of TB disease, previous HWS visits (the last one year), the number of days spent at the HWS, and shared living space (room) at the HWSs, were recorded.

### 2.5. Culture and Identification Test

Sputum specimens from attendees with PTB symptoms were obtained using sterile and leak-proof collection containers [18]. The sputum specimen was placed in an icebox and delivered to the Amhara Public Health Institute, a regional public health referral laboratory center, Bahir Dar, Ethiopia [18]. Sputum specimens were prepared and inoculated into a Löwenstein–Jensen (LJ) culture medium following standard laboratory procedures. Ziehl–Neelsen (ZN) staining was done to confirm all LJ culture-positive isolates [22]. The MPT64 antigen test (Capilia TB-Neo, TAUNS Laboratories, Inc., Japan) was used to differentiate MTB complex species from non-TB mycobacteria (NTM) [23].

### 2.6. Specimen Preparation

After subculturing, suspensions were prepared from LJ-positive specimens and transferred into 1.5 ml of PrimeStore Molecular Transport Medium (PS-MTM; Longhorn Vaccine and Diagnostics, San Antonio, Texas, USA). Preparing suspensions of MTB colonies from LJ culture media depended on the culture state [24]. In brief, for intact slopes, colonies were gently scraped off using an inoculation loop and suspended (washed down) in 1 ml of sterile water in the original culture bottle. After pipetting off the suspension, it was transferred into a 1.5-ml Eppendorf tube and then transferred to the 1.5 ml PS-MTM [24]. Mycobacterial suspensions prepared in PS-MTMP tubes were transported to South Africa by air at ambient temperature for other genotyping procedures.

### 2.7. DNA Extraction

The MTB DNA was extracted from all 122 LJ-positive isolates using the PrimeXtract™ kit (Longhorn Vaccines and Diagnostics, San Antonio, TX, USA) following the manufacturer's instructions [25]. Briefly, 200 *μ*L of 100% ethanol, 200 *μ*L of lysis buffer, and 200 *μ*L of MTB inoculum (preserved in PS-MTM) were transferred into a 1.5-mL microcentrifuge tube, then vortexed and centrifuged. The entire supernatant was transferred to a microextraction column and centrifuged at 13,000 rpm for 1 minute, and the flow-through material was discarded. Wash buffer 1 (500 *μ*L) was applied to the extraction column and centrifuged at 13,000 rpm for 1 minute, followed by further addition of wash buffer 2 (500 *μ*L) to the extraction column and subsequent centrifuging as described above, discarding the flow-through material. Then, DNA was eluted by 1 minute of centrifugation at 13,000 rpm using 50 *μ*L of preheated (60–70°C) elution solution [25]. For future use, the extracted MTB DNA was preserved at −20°C. The concentration and quality of extracted genomic DNA were assessed using a spectrophotometer at the optimal densities of 280 nm and 260 nm [18].

### 2.8. Drug Susceptibility Testing

Following MTB genomic DNA extraction using the PrimeXtract™ kit instructions [25], the second-generation line probe assays (LPAs), MTBDR*plus* VER2.0 (to detect RIF and INH resistance), and MTBDR*sl* VER2.0 kit strips (to detect FLQs and aminoglycosides/peptide resistance) were performed following the manufacturer's protocol (Hain Lifescience GmBH, Nehren, Germany) [26, 27]. All INH and RIF-resistant TB isolates were tested using MTBDR*sl* VER 2.0 for detecting FLQs and second-line injectable drugs (SLIDs)-resistant isolates. For each run of LPAs, MTB strains H37Rv susceptible to all anti-TB drugs tested and molecular-grade water were used as a positive and negative control, respectively.

### 2.9. Prospective Follow-Up Study

A prospective follow-up study on 438 PTB-symptomatic individuals with culture-negative test results while at HWSs was done to determine the prevalence of developing active TB disease subsequent to residing at HWS. The duration of follow-up was 12 months, starting on the date that a negative culture result was obtained. TB status was confirmed via telephone or in-person contact, and any reports of active TB were confirmed by reviewing the patient's medical records at the diagnosing public healthcare facility. The date of diagnosis, the diagnostic method (acid-fast bacilli (AFB) smear microscopy, GeneXpert®MTB/RIF assay, LPAs, or other diagnoses, like chest X-rays, and clinical diagnoses), the diagnostic public healthcare facility, and the location (zone) were recorded from participants who reported developing active TB disease during the follow-up period.

### 2.10. Data Analysis

Laboratory test results and socio-demographic data were recorded in a Microsoft Excel spreadsheet, coded, and entered into STATA 17 (STATA; StataCorp) for analysis. The data were checked for completeness and consistency by running the frequencies of each variable. The results were summarized using descriptive statistics. Bivariate and multivariate logistic regression analysis models were employed to identify associated risk factors for culture-positive TB and developing active TB disease post-residency at the HWSs. Predictors having a *p* < 0.2 in the bivariable analysis were included in the multivariate analysis. In multivariate logistic regression analysis, the odds ratio (OR) and 95% confidence intervals were retrieved and variables with a *p* value of less than 0.05 were considered statistically significant.

### 2.11. Research Ethics

The Human Research Ethics Committee of the Faculty of Health Sciences, University of Pretoria, South Africa (Ethics Ref. No: 600/2018), and the Ethiopian National Research Ethics Review Committee (Ethics Ref. No: SHE/SM/14.4/708/2019) approved the study. Moreover, a signed official permission letter was sought from the Orthodox Tewahedo Church, Patriarchate Head Office, Addis Ababa, Ethiopia (Ref. No. 2478/6275/2011). A verbal or written consent declaration with details about the study was given or explained to participants and signed, and the study was conducted following the Helsinki Declaration. Participants who tested positive for PTB were transferred/linked to a nearby public healthcare facility for treatment and further patient management.

## 3. Results

### 3.1. Socio-Demographic Profiles of Participants

A total of 10,313 HWS attendees were screened for PTB-suggestive symptoms, and 560 (5.4%) were found to have PTB symptoms and participated. Of 560 participants, 308 (55.0%) were males and 263 (47.0%) were between the ages of 18 and 33 with a median age of 35 years. Most of the study participants (356; 63.6%) were married, whereas 302 (53.9%) were rural residents (Table 1).

### 3.2. Prevalence of Culture-Positive Pulmonary Tuberculosis

Of 560 PTB-symptomatic participants, 122 (21.8%) (95 CI: 18.4–25.2%) were culture-positive, resulting in a point prevalence of 1,183 per 100,000 attendees. The proportion of males and females with culture-positive PTB was nearly the same (21.8% each). The majority of bacteriologically confirmed cases (76/302; 25.2%) were rural residents. Moreover, participants aged 18–33 years (75/263; 28.5%) had a higher rate of culture-positive PTB (Table 2 and Table S2).

### 3.3. Risk Factors for Culture-Positive Pulmonary Tuberculosis

The bivariate logistic regression analysis revealed that participants aged 34–49 years ((crude odds ratio (cOR) 0.44; 95CI: 0.28–0.70)), and rural residents (cOR 1.55; 95CI: 1.03–2.34) were statistically associated with culture-positive PTB. Additionally, the analysis revealed that few independent variables were statistically associated with culture-positive PTB (*p* < 0.05), including a history of TB disease, contact with chronic coughers or active TB patients, having had close contact with a family member who has TB, the number of days (>21) spent at HWS, and sharing living spaces (rooms) at HWS (Table S3).

In the final multivariate logistic regression model, place of residence, marital status, family size per household, and sharing a living space (rooms) at HWSs were positively associated with culture-positive PTB. Rural residents were two times more likely to develop culture-positive PTB compared to urban residents (aOR 2.65; 95CI: 1.38–5.10). Married participants were more likely to have culture-positive PTB than single participants (aOR 2.43; 95CI: 1.28–4.63). On the other hand, participants with more than five family members per household were 1.84 times more likely to have culture-positive PTB than those with less than five (aOR 1.84; 95CI:1.04–3.24). Besides, sharing a living space at HWS increased the risk of developing culture-positive PTB by tenfold (aOR 10.57; 95CI: 3.60–31.13) (Table 3).

### 3.4. Drug Resistance Profiles of Mycobacterium tuberculosis Isolates

Of 122 isolates tested, 83.6% (102) were susceptible to both RIF and INH, while 16.4% (20) were resistant to RIF and/or INH. Any INH and RIF-resistant TB was detected in 16.4% (20) and 12.3% (15) isolates, respectively. Multidrug-resistant TB (MDR-TB) (resistant to both RIF and INH) was found in 12.3% (15) of culture-positive cases, five (4.1%) of which were fluoroquinolones- (FLQs-) resistant TB isolates. These five FLQs-resistant strains were identified at the South Wello zone study site. Furthermore, 7.1% (3/42 retreated cases) of RIF-resistant/MDR (RR/MDR) TB isolates were detected in previously treated TB cases (Table 4).

### 3.5. Risk Factors Associated with Drug Resistance

The multivariate logistic regression analysis revealed that a history of TB disease and study area has a significant association with the occurrence of some form of drug-resistant TB. Attendees who had a history of TB disease were nine times more likely to suffer from drug-resistant TB as compared with other HWS attendees who had no history of TB (aOR 9.22; 95 CI: 1.55–54.82). On the other hand, participants who attended HWS in the South Wello zone were three times more likely to develop any drug-resistant TB as compared with those who attended HWS in Central Gondar (aOR 3.06; 95 CI: 0.21–6.72). Similarly, the analysis revealed that attendees' occupations and study areas have a significant association with the occurrence of MDR-TB. Thus, farmers and housewives were nine (aOR 9.78; 95 CI: 1.55–61.59) and fifteen (aOR 15.68; 95 CI: 1.46–168.10) times more likely to develop MDR-TB as compared with students, respectively. Moreover, participants who attended HWS in the South Wello zone were twice as likely to develop MDR-TB as compared to those who attended HWS in Central Gondar (aOR 2.08; 95 CI: 0.31–3.92) (Table 5).

In addition, the proportion of any drug-resistant and MDR-TB in each study zone was estimated based on the participant's age category and found that participants aged 18–33 years appeared to be the most affected. Thus, attendees aged 18–33 years who attended HWS in the South Wello zone had the highest rate of any drug-resistant TB, with a rate of 47.1% ± 12.1SE (95 CI: 24.3–71.1%), and MDR-TB with a rate of 58.3% ± 14.2SE (95 CI: 28.5–83.1%) (Table S4). On the other hand, the logistic regression analysis showed that the odds of developing any drug-resistant TB and MDR-TB were 14.54 (95 CI: 1.65–128.44) and 12.00 (95% CI: 1.35–106.80) times higher in the South Wello zone compared to the North Wello zone (Table S5).

### 3.6. The Prospective Follow-Up Study Results

A prospective follow-up study on 438 PTB-symptomatic individuals with culture-negative tests while at HWS was done to determine the proportion of developing active TB disease subsequent to residing at HWS. The duration of follow-up was 12 months, starting on the date that a negative culture result was obtained. Among 438 participants who were on follow-up, 30 (6.8%) (95 CI 4.4–9.4%) developed active TB disease post-residency at the HWS. Male participants and those who were over 50 years of age had a great share of developing active TB disease post-residency at the HWSs, with a proportion of 5.3% (*n* = 23) and 3.3% (*n* = 14), respectively (Table S6).

### 3.7. Risk Factors for Developing Active TB Disease Post-Exposure to HWS

The multivariate logistic regression analysis revealed that gender and educational status of participants were associated risk factors for developing active TB disease post-residency at the HWSs. Thus, the odds of developing active TB disease were eight times higher among females (aOR 8.43; 95 CI: 1.90–36.70), whereas it was sixfold higher among those who cannot read and write (aOR 6.09; 95 CI: 1.25–29.55). Furthermore, the odds of developing active TB disease were higher among participants who had a history of contact with TB patients in their family members (aOR 25.20; 95 CI: 3.01–206.50), shared living space at HWS (aOR 37.19; 95 CI: 2.46–561.20), and those who had recently been hospitalized (aOR 14.50; 95 CI: 1.16–180.90) compared to their counterparts (Table S7).

## 4. Discussion

In Ethiopia, HWSs are traditional places of healing where people travel all over to seek the restorative benefits of holy water blessed by Orthodox priests [13, 16, 19]. In the study region, people use faith-based therapy with spiritual holy water for different diseases [13, 16, 17]. Although studies have shown that TB patients seek treatment from traditional healers and HWSs, the burden of TB among HWS attendees in Ethiopia has not been thoroughly studied.

In this study, the prevalence of culture-positive PTB was 21.8%. Thus, the point prevalence was 1,183 per 100,000 attendees, which was 4.3 times higher than a national TB prevalence study in Ethiopia, which reported 277 per 100,000 bacteriologically confirmed TB cases and 108 per 100,000 smear-positive TB cases [28]. Similarly, our finding was 1.5-fold higher than a previous study that reported 795 per 100,000 HWS attendees had TB in the study region [13]. The same study reported that the prevalence of smear-positive PTB among adult HWS attendees was 7.4-fold higher than Ethiopia's national TB prevalence [13]. This discrepancy may be due to the study population and laboratory diagnosis methods. The national TB prevalence survey report in Ethiopia excluded congregate settings and high-risk groups found in the HWSs [28]. On the other hand, Derseh and his colleagues used sputum smear microscopy, which has low sensitivity for TB detection, resulting in a low-prevalence finding report [13]. The high prevalence of culture-positive PTB at HWSs may be due to overcrowding, close contact, inadequate ventilation of shared living spaces, and long stays, which increase exposure and TB transmission [13]. People who visit HWS choose to treat their diseases religiously, including TB and HIV/AIDS, and perceive spiritual HWS as their best treatment choice [13, 16, 18]. In addition, people in rural areas and poor communities lack knowledge of TB and often misinterpret TB symptoms as signs of other diseases [14]. Another possible explanation for the high prevalence of PTB in our study and the report of a previous study of a similar nature [13] could be due to study setting selection biases since persons with TB symptoms are more likely to visit HWS to treat the disease spiritually [18, 29, 30]. The prevalence of culture-positive PTB (21.8%) in the current study was also higher than that found in other high-risk settings in Ethiopia, including prisons, homeless shelters, and university students [31–38]. The difference may be due to the fact that these studies used sputum smear microscopy, whereas we used conventional culture methods, which are more sensitive and specific for TB diagnosis. Another probable explanation is that everyone who attends HWSs is prone to TB and other infectious diseases due to their various health conditions. In addition, the availability of TB diagnosis and treatment services in prisons and universities may enable the early diagnosis and treatment of TB cases. Our study result was also higher than community-based studies in Southern Ethiopia's rural districts, which reported 3.0 to 6.3% [39–41]; Amhara region (3.8 to 4.9%) [11, 12]; Oromia region (7.6%) [42]; Tigray, Northern Ethiopia (8.6%) [43]; and central Ethiopia, Addis Ababa (13.3%) [44]. The difference may be due to differences in the study populations (subnational versus HWS attendees), TB diagnostic methods (sputum smear microscopy versus culture technique), the study period, and study setting selection biases.

The current study found that 18–33 years of age participants had the highest rate of culture-positive PTB. This may be because 47.0% of study participants were 18–33 years of age. Another possible explanation is that young people are more likely to regularly attend HWS and are hence likely to be exposed to TB infection. Most HWSs are in remote places, making it difficult for older adults to attend regularly. A similar study found that 68.6% of spiritual HWS attendees were 15–45 years of age [13]. The burden of TB among young individuals has major health consequences, as these individuals are economically active, and their travel for employment and high social interactions within the community exacerbate TB transmission in the general population [45].

This study found that rural residents are more likely to have culture-positive TB than urban residents. This was consistent with other studies' reports [46, 47]. According to global statistics, urban areas have a higher TB burden; however, TB is also prevalent among rural inhabitants in countries where a large portion of the population resides in rural areas and has a low income [48]. Due to inadequate public healthcare facilities, poor TB services, individuals' poor healthcare-seeking behavior, and a lack of knowledge and information about TB, early diagnosis and treatment are especially difficult in rural areas [40]. On the other hand, due to travel costs, fear of stigma, and other sociocultural and socioeconomic factors, people with TB in rural areas are unable to access healthcare facilities for early diagnosis and treatment [14, 15]. However, further research is required to investigate this interaction.

The majority of participants with culture-positive TB in the current study were married. This was consistent with a previous study [48]. In contrast to our findings, a few studies have revealed that unmarried people have a higher risk of TB infection than married people [46, 49, 50]. This might be because single people have a different lifestyle than married individuals. However, further study is necessary to better understand the factors that influence marital status as a predictor of active TB.

Household size (>5 family members) was statistically associated with culture-positive TB in the current study. This was consistence with a previous similar study [13] and institution-based studies in southeast Ethiopia [49, 51]. This may be because poverty, malnutrition, and overcrowded living conditions all increase the risk of TB transmission [5]. Since TB is mainly transmitted indoors, having a large family size per household results in overcrowding and increases the risk of TB transmissions. Another possible explanation is that people who live in rural areas and have many family members are more likely to be of lower socioeconomic status.

In our study, participants who shared living space at HWSs were tenfold more likely to have culture-positive PTB. This is because rooms at the HWSs are built as temporary waiting spaces, and they are very small, overcrowded, and poorly ventilated, which can intensify the risk of TB transmission [13, 18, 52]. The overcrowded and poorly ventilated waiting rooms at the HWS, prolonged stays at the HWS, and poor healthcare-seeking behaviors of individuals worsen the active transmission of TB at the HWS. Our findings were comparable to those of prior studies [32, 34, 35]. Congregate settings, such as HWSs, are places where people live together, and it is common for people to share living space [13, 18, 52]. Hence, poor living conditions and overcrowding in the shared room can increase TB transmission.

Drug-resistant TB (DR-TB) threatens national TB prevention and care efforts. In the current study, the prevalence of any DR-TB (resistance to RIF and/or INH) was 16.4%, indicating that it is a significant concern among HWS attendees. This is likely because many people with TB (infected with DR-TB strains) attended HWSs, resulting in DR-TB transmission [13, 18, 52]. This is comparable with previous reports from the study region (10.3 to 20.2%) [53–59], and other parts of Ethiopia (11.1 to 18.4%) [60–65]. However, our finding was lower than the finding reported in Ethiopia [66–69]. The discrepancy may be due to the study population (PTB-symptomatic HWS attendees versus TB patients who visited public healthcare facilities), and different drug susceptibility testing (DST) methods (phenotypic and/or PCR-based), which have varied sensitivity for diagnosing DR-TB strains.

Moreover, our study found 12.3% MDR-TB (resistance to both RIF and INH), suggesting that MDR-TB is also a major concern in these high-risk groups. Furthermore, 7.1% (3/42 retreated cases) of RR/MDR-TB isolates were identified in previously treated, while 15% (12/80) were isolated from newly diagnosed TB cases. Our result was higher than earlier studies in the study region (1.0 to 8.4%) [53, 54, 58, 59, 70, 71] and elsewhere in Ethiopia (1.2% to 8.3%) [64, 67–69, 72, 73]. It was also higher than 4.4% in the Ethiopian national survey [66], 0.71% in a recent national report on MDR-TB among new cases [1], and prison settings in Ethiopia (9.5%) [74]. However, it was comparable to previous studies from central Ethiopia, Addis Ababa [61, 75], Eastern Ethiopia [63], and a recent Ethiopian national report on MDR-TB among retreated TB patients (12.0%) [1]. The discrepancy may be due to differences in study populations, study settings, and DST methods used to diagnose MDR-TB. The high prevalence of DR-TB, particularly MDR-TB, among HWS attendees in the study region indicated that many TB patients, including those infected with DR-TB strains, attended the sites and that DR-TB strain transmission is common. However, molecular epidemiology studies are necessary to understand resistant TB strain transmission in HWS attendees and the community.

In this study, 4.1% of isolates were FLQs-resistant. Interestingly, all five FLQs-resistant and/or pre-XDR-TB isolates were identified at the South Wello zone. This suggests that these pre-XDR-TB strains may be circulating in this study site and have recently disseminated, although a molecular epidemiology analysis with strong discriminatory power would be required to confirm genotypic similarities, and recent transmission [18, 52]. Consistent with our result, an earlier study conducted in the same study region found that 5.7% of TB patients had pre-XDR-TB strains [7]. Similarly, a multicenter study revealed that the prevalence of pre-XDR-TB in Ethiopia was 5.0% [76]. However, both studies and ours had quite different study populations. Shibabaw and his colleagues included all RIF-resistant or MDR-TB patients who attended MDR-TB treatment centers before starting anti-TB therapy [7], while Dagne and his colleagues included new and retreated TB cases who attended TB treatment centers in different Ethiopian settings [76]. Thus, our study demonstrated that DR-TB, particularly MDR-TB, and pre-XDR-TB, among PTB-symptomatic HWS attendees in Ethiopia is a major issue that necessitates urgent prevention intervention measures and more studies on the same high-risk groups.

In the present study, participants between the ages of 18 and 33 years appeared to be at the highest risk of any DR-TB and MDR-TB infection, with rates of 47.1% and 58.3%, respectively. Although the comparison is difficult since different studies use different age cutoff points, an earlier study in the same study region found a strong link between anti-TB drug resistance and the age range of 25 to 34 years [58]. Similarly, a study conducted in South Africa revealed a strong link between DR-TB and the age groups 35 to 54 years and over 55 years [77]. Young age groups' increased exposure to the external environment, high-risk behavior, high workload, and broad range of mobility might contribute to TB and DR-TB infection. The high rate of DR-TB among younger folks has major health repercussions, as these individuals are economically active, and their travel for employment and high social contacts within the community exacerbates TB transmission in the general population [45].

In addition, in the current study, the South Wello zone study area appears to have the highest rate of any DR-TB and MDR-TB, with the odds of 14.5- and 12.0-fold higher than other study sites, respectively. Interestingly, five FLQs-resistant and/or pre-XDR-TB strains were identified at the South Wello zone study site. This could be because DR-TB is prominent in this study area and there is recent TB transmission, although further molecular epidemiology analysis with strong discriminatory power would be required to confirm genotypic similarities and transmission patterns of the resistant strains [18, 52].

In the prospective follow-up study, a significant proportion of participants reported the development of active TB disease following exposure to HWS. Although our prospective follow-up method has limitations (unable to collect specimens for further laboratory confirmation), it suggests that HWS is a high-risk setting for TB transmission. Thus, these individuals can spread the disease to the community and their household members. We found that the attendee's educational status and sex, having had close contact with active TB patients in their family members, sharing living space at HWS, and recent hospitalization were potential risk factors for developing active TB disease post-residency at the HWS. TB transmission will be enhanced in environments where social mixing is more likely (along with overcrowding). Similarly, factors that prolong an infectious patient's exposure time will enhance TB transmission to other individuals [78].

### 4.1. Limitations

There were a few limitations to our study. First, there is a population selection bias and likely information bias when using self-reported data on risk factors. Second, the confirmatory tests were not done on those who reported active TB progression post-residency at the HWSs. Besides, in the follow-up study cohort, a comparison group of people who never attended or had no HWS exposure is crucial to determine the level of risk of HWS exposure to acquiring active TB post-residency at the HWS. Third, due to financial constraints, we were unable to perform BD BACTEC MGIT 960 for the initial MTB isolation process, and no phenotypic DST (pDST) was done on these isolates because liquid culture techniques, especially MGIT 960, have a significant advantage over solid culture techniques in terms of turnaround time and better recovery of MTB isolates. Last, further molecular epidemiology analysis would be warranted to confirm the transmission patterns of TB strains among HWS attendees.

## 5. Conclusions

Given that the prevalence of PTB among HWS attendees in this study population was seen to be higher than in the general population, hence, proactive preventive measures are recommended. The current study revealed that rural residents, being married, having >5 family members, and sharing a living space at HWSs were predictors of culture-positive PTB. The follow-up study also revealed that a higher proportion of attendees developed active TB disease post-residency at the HWS. Furthermore, the study showed a higher rate of DR-TB, especially MDR-TB, and pre-XDR-TB, among HWS attendees. Participants aged 18–33 years and the South Wello zone study site appeared to be more affected by DR-TB strains. Thus, regional and national TB prevention and control programs should recognize HWSs as high-risk settings for TB transmission and implement regular systematic TB screening, detection of DR-TB strains, and routine drug resistance surveillance among HWS attendees. Robust collaboration between the Ethiopian Orthodox Tewahedo Church and the regional and national TB control program is essential to develop locally appropriate, culturally accepted, and effective policy interventions to halt TB transmission among HWS attendees and the community.

## Figures and Tables

**Table 1:** Socio-demographic characteristics of PTB-symptomatic HWS attendees (*n* = 560).

Socio-demographic characteristics		Frequency, *n* (%)
Sex	Male	308 (55.0)
	Female	252 (45.0)

Age (year)	18–33	263 (47.0)
	34–49	208 (37.1)
	≥50	89 (15.9)

Residence	Urban	258 (46.1)
	Rural	302 (53.9)

Marital status	Married	356 (63.6)
	Single^*∗*^	204 (36.4)

Educational status	Can't read and write	256 (45.7)
	Primary school	165 (29.5)
	Secondary school and above	139 (24.8)

Household size	1–5	294 (52.5)
	>5	266 (47.5)

Occupation	Farmer	235 (42.0)
	Employed^a^	24 (4.3)
	Unemployed^b^	117 (20.9)
	Housewife	94 (16.8)
	Students and others^*∗∗*^	90 (16.1)

*Notes*. ^*∗*^Single, divorced, and widowed; ^*∗∗*^religious leaders and deacons; HWS: holy water site; PTB: pulmonary tuberculosis; ^a^construction worker, administrative worker, healthcare worker, public transport worker; ^b^businessman, trader, daily laborer.

**Table 2:** Proportion of bacteriologically confirmed PTB with socio-demographic characteristics of participants (*n* = 560).

Socio-demographic characteristics		Prevalence of culture-positive PTB		*p* value
		Positive, *n* (%)	Negative, *n* (%)	
Sex	Male	67 (21.8)	241 (78.2)	1.00
	Female	55 (21.8)	197 (78.2)	

Age group (year)	18–33	75 (28.5)	188 (71.5)	0.001
	34–49	31 (14.9)	177 (85.1)	
	≥50	16 (18.0)	73 (82.0)	

Residence	Urban	46 (17.8)	212 (82.2)	0.040
	Rural	76 (25.2)	226 (74.8)	

Marital status	Married	85 (23.9)	271 (76.1)	0.136
	Single^*∗*^	37 (18.1)	167 (81.9)	

Educational status	Can't read and write	59 (23.0)	197 (77.0)	0.396
	Primary school	30 (18.2)	135 (81.8)	
	Secondary school and above	33 (23.7)	106 (76.3)	

Household size	1–5	57 (19.4)	237 (80.6)	0.153
	>5	65 (24.4)	201 (75.6)	

Occupation	Farmer	45 (19.1)	190 (80.9)	0.495
	Employed^a^	6 (25.0)	18 (75.0)	
	Unemployed^b^	23 (19.7)	94 (80.3)	
	Housewife	24 (25.5)	70 (74.5)	
	Students and others^*∗∗*^	24 (26.7)	66 (73.3)	

*Notes*. ^*∗*^Single, divorced, and widowed; ^*∗∗*^religious leaders and deacons; PTB: pulmonary tuberculosis; ^a^construction worker, administrative worker, healthcare worker, public transport worker; ^b^businessman, trader, daily laborer.

**Table 3:** Multivariate logistic regression analysis of potential associated risk factors for culture-positive TB among PTB-symptomatic HWS attendees (*n* = 560).

Variables		Culture-positive PTB		aOR (95% CI)	*p* value
		Positive (*n*)	Negative (*n*)		
Residence	Urban	46	212	Ref	0.003
	Rural	76	226	2.65 (1.38–5.10)	

Marital status	Married	85	271	2.43 (1.28–4.63)	0.007
	Single^*∗*^	37	167	Ref	

Household size	1–5	57	237	Ref	0.036
	>5	65	201	1.84 (1.04–3.24)	

Sharing drinking cups at the HWS	Yes	91	268	10.59 (3.60–31.13)	<0.001
	No	31	170	Ref	

Sharing a living space at the HWS	Yes	118	320	10.57 (3.60–31.13)	<0.001
	No	4	118	Ref	

Do you know your HIV status?	Yes	34	179	Ref	0.001
	No	88	259	2.69 (1.50–4.84)	

*Notes*. ^*∗*^Single, divorced, and widowed; aOR: adjusted odds ratio; CI: confidence interval; HIV: human immunodeficiency virus; HWS: holy water sites; PTB: pulmonary tuberculosis; ref: reference.

**Table 4:** The proportion of drug-resistant TB isolates and characteristics of study participants (*n* = 122).

Characteristics		*N*	Anti-TB drug resistance patterns			
			INH^r^, *n* (%)	RIF^r^, *n* (%)	RIF^r^/MDR-TB, *n* (%)	FLQs-resistant, *n* (%)
Sex	Male	67	14 (20.9)	9 (13.4)	9 (13.4)	2 (3.0)
	Female	55	6 (10.9)	6 (10.9)	6 (10.9)	3 (5.5)

Age (year)	18–33	75	17 (22.7)	12 (16.0)	12 (16.0)	5 (6.7)
	34–49	31	0 (0.0)	0 (0.0)	0 (0.0)	0 (0.0)
	≥50	16	3 (18.8)	3 (18.8)	3 (18.8)	0 (0.0)

Residence	Urban	46	10 (21.7)	7 (15.2)	7 (15.2)	2 (4.3)
	Rural	76	10 (13.2)	8 (10.5)	8 (10.5)	3 (3.9)

Marital status	Married	85	11 (12.9)	7 (8.2)	7 (8.2)	2 (2.4)
	Single^*∗*^	37	9 (24.3)	8 (21.6)	8 (21.6)	3 (8.1)

Educational status	Can't read and write	59	6 (10.2)	4 (6.8)	4 (6.8)	0 (0.0)
	Primary school	30	8 (26.7)	7 (23.3)	7 (23.3)	4 (13.3)
	Secondary school and above	33	6 (18.2)	4 (12.1)	4 (12.1)	1 (3.0)

Household size	1–5	57	13 (22.8)	9 (15.8)	9 (15.8)	3 (5.3)
	>5	65	7 (10.8)	6 (9.2)	6 (9.2)	2 (3.1)

Occupation	Farmer	45	4 (8.9)	2 (4.4)	2 (4.4)	1 (2.2)
	Employed^a^	6	3 (50.0)	2 (33.3)	2 (33.3)	1 (16.7)
	Unemployed^b^	23	5 (21.7)	3 (13.0)	3 (13.0)	1 (4.3)
	Housewife	24	1 (4.2)	1 (4.2)	1 (4.2)	0 (0.0)
	Students and others^*∗∗*^	24	7 (29.2)	7 (29.2)	7 (29.2)	2 (8.3)

Study area (zone)	North Wello	22	1 (4.5)	1 (4.5)	1 (4.5)	0 (0.0)
	South Wello	22	9 (40.9)	8 (36.4)	8 (36.4)	5 (22.7)
	North Shewa	33	3 (9.1)	3 (9.1)	3 (9.1)	0 (0.0)
	South Gondar	28	6 (21.4)	2 (7.1)	2 (7.1)	0 (0.0)
	Central Gondar and others^*∗∗∗*^	17	1 (5.9)	1 (5.9)	1 (5.9)	0 (0.0)

Types of PTB cases	Previously treated	42	3 (7.1)	3 (7.1)	3 (7.1)	1 (2.4)
	Newly diagnosed	80	17 (21.3)	12 (15.0)	12 (15.0)	4 (5.0)

*Notes*. ^*∗*^Single, divorced, and widowed; ^*∗∗*^others: religious leaders and deacons; ^*∗∗∗*^others: Awi zone, West Gojjam, East Gojjam, and Wag-Hamra FLQs: fluoroquinolones; HWS: holy water site; INH^r^: isoniazid resistance; MDR: multidrug-resistant; *N*: total number of culture-positive cases; PTB: pulmonary tuberculosis; RIF^r^: rifampicin resistance; TB: tuberculosis; ^a^construction worker, administrative worker, healthcare worker, public transport worker; ^b^businessman, trader, daily laborer.

**Table 5:** Factors associated with any drug-resistant TB (RIF^r^ and/or INH^r^) and RIF^r^/MDR-TB.

Variables		*N*	Any drug resistance (RIF^r^ and/or INH^r^)		aOR (95% CI)	*p* value
			Yes, *n* (%)	No, *n* (%)		

History of TB disease	Yes	41	2 (4.9)	39 (95.1)	9.22 (1.55–54.82)	0.015
	No	81	18 (22.2)	63 (77.8)	Ref	
Occupation	Employed	6	3 (50.0)	3 (50.0)	0.74 (0.06–9.27)	0.817
	Unemployed	23	5 (1.7)	18 (78.3)	1.19 (0.26–5.36)	0.823
	Farmer	45	4 (8.9)	41 (91.1)	4.57 (0.99–21.19)	0.052
	Housewife	24	1 (4.2)	23 (95.8)	10.18 (0.95–109.55)	0.056
	Student and others^*∗*^	24	7 (29.2)	17 (70.8)	Ref	
The study area (zone)	North Wello	22	1 (4.5)	21 (95.5)	0.96 (0.05–20.16)	0.980
	South Wello	22	9 (40.9)	13 (59.1)	3.06 (0.21–6.72)	0.026
	North Shewa	33	3 (9.1)	30 (90.9)	0.82 (0.07–9.36)	0.872
	South Gondar	28	6 (21.4)	22 (78.6)	0.23 (0.02–2.34)	0.212
	Central Gondar and others^*∗∗*^	17	1 (5.9)	16 (94.1)	Ref	

			RIF^r^/MDR-TB		aOR (95% CI)	*p* value
			Yes, *n* (%)	No, *n* (%)		

Marital status	Married	85	7 (8.2)	78 (91.8)	—	—
	Single^*∗∗∗*^	37	8 (21.6)	29 (78.4)	Ref	—
Occupation	Employed^a^	6	2 (33.3)	4 (66.6)	2.84 (0.27–29.59)	0.383
	Unemployed^b^	23	3 (13.0)	20 (87.0)	3.42 (0.62–18.84)	0.157
	Farmer	45	2 (4.4)	43 (95.6)	9.78 (1.55–61.59)	0.015
	Housewife	24	1 (4.2)	23 (95.8)	15.68 (1.46–168.10)	0.023
	Student and others^*∗*^	24	7 (29.2)	17 (70.8)	Ref	
Study area (zone)	North Wello	22	1 (4.5)	21 (95.5)	0.83 (0.04–16.71)	0.901
	South Wello	22	8 (36.4)	14 (63.6)	2.08 (0.31–3.92)	0.043
	North Shewa	33	3 (9.1)	30 (90.9)	0.61 (0.05–6.96)	0.688
	South Gondar	28	2 (7.1)	26 (92.9)	0.85 (0.07–11.13)	0.901
	Central Gondar and others^*∗∗*^	17	1 (5.9)	16 (94.1)	Ref	

*Notes*. ^*∗*^Others: religious leaders and deacons; ^*∗∗*^others: Awi, West Gojjam, Wag-Hamra, East Gojjam; ^*∗∗∗*^single, widowed, and divorced; aOR: adjusted odds ratio; CI: confidence interval; INH^r^: isoniazid resistance; ref: reference; RIF^r^/MDR-TB: rifampicin-resistant/multidrug-resistant tuberculosis; RIF^r^: rifampicin resistance; TB: tuberculosis; ^a^construction worker, administrative worker, healthcare worker, public transport worker; ^b^businessman, trader, daily laborer.

## Data Availability

The data sets analyzed during this study are available from the corresponding author upon reasonable request.

## Supplementary Materials

Supplementary Materials Table S1 illustrates the administrative zones that were included during the study, the spiritual holy water sites that were selected from each administrative zone, the total number of attendees who were screened for pulmonary TB (PTB) symptoms, the number of attendees who had PTB-suggestive symptoms, the number of bacteriologically confirmed cases, and the number of individuals who were Löwenstein–Jensen culture-negative test result. Table S2 shows the proportion of PTB positivity by gender and age group. The proportion estimation analysis revealed that the prevalence of culture-positive PTB was nearly equal for males and females (21.8% ± 2.35SE Vs 21.8% ± 2.6SE). Also, participants aged 18 to 33 years had a higher proportion of culture-positive PTB (28.5% ± 2.8SE). Table S3 summarizes the bivariate logistic regression analysis of socio-demographic characteristics of participants and associated risk factors for culture-positive PTB. The analysis revealed that participants aged 34–49 years and rural residents were statistically associated with culture-positive PTB (*p* < 0.05). Furthermore, the analysis revealed that few independent variables were statistically associated with culture-positive PTB (*p* ≤ 0.01), including a history of TB disease, contact with chronic coughers or active TB patients, having had close contact with a family member who had TB, the number of days spent (>21) at HWS, and sharing living spaces at HWS. In Table S4, we compute the proportion of any drug-resistant TB and MDR-TB among each age group of participants in each study area. This allows us to assess the extent to which each age group appears to be affected by any drug-resistant TB and MDR-TB strains. Similarly, as illustrated in Table S5, we used a logistic regression analysis model to determine the odds of developing any drug-resistant TB and MDR-TB among participants in each study site. Tables S6 and S7 provide detailed data about participants who had culture-negative test results while at HWS. Table S6 illustrates the profiles of participants and the proportion of developing active TB disease post-residency at the HWS among those who had culture-negative results while at HWS. Besides, Table S7 shows the bivariate and multivariate logistic regression analysis of the socio-demographic characteristics of participants and associated factors among those who reported contracting active TB disease post-exposure to HWS.
