# Supplementary material for: Spiritual Holy Water Sites in Ethiopia: Unrecognized High-Risk Settings for Transmission of Pulmonary Tuberculosis
Source: Int J Microbiol. 2024 Apr 8;2024:3132498. doi: 10.1155/2024/3132498 (PMC11018379; doi:10.1155/2024/3132498)
Supplement: Supplementary Materials — Table S1 illustrates the administrative zones that were included during the study, the spiritual holy water sites that were selected from each administrative zone, the total number of attendees who were screened for pulmonary TB (PTB) symptoms, the number of attendees who had PTB-suggestive symptoms, the number of bacteriologically confirmed cases, and the number of individuals who were Löwenstein–Jensen culture-negative test result. Table S2 shows the proportion of PTB positivity by gender and age group. The proportion estimation analysis revealed that the prevalence of culture-positive PTB was nearly equal for males and females (21.8% ± 2.35SE Vs 21.8% ± 2.6SE). Also, participants aged 18 to 33 years had a higher proportion of culture-positive PTB (28.5% ± 2.8SE). Table S3 summarizes the bivariate logistic regression analysis of socio-demographic characteristics of participants and associated risk factors for culture-positive PTB. The analysis revealed that participants aged 34–49 years and rural residents were statistically associated with culture-positive PTB (p < 0.05). Furthermore, the analysis revealed that few independent variables were statistically associated with culture-positive PTB (p ≤ 0.01), including a history of TB disease, contact with chronic coughers or active TB patients, having had close contact with a family member who had TB, the number of days spent (>21) at HWS, and sharing living spaces at HWS. In Table S4, we compute the proportion of any drug-resistant TB and MDR-TB among each age group of participants in each study area. This allows us to assess the extent to which each age group appears to be affected by any drug-resistant TB and MDR-TB strains. Similarly, as illustrated in Table S5, we used a logistic regression analysis model to determine the odds of developing any drug-resistant TB and MDR-TB among participants in each study site. Tables S6 and S7 provide detailed data about participants who had culture-negative test results while a [file 3132498.f1.docx]

**Supplementary Files:**

**Descriptions:**

**Table S1** illustrates the administrative zones that were included during the study, the spiritual holy water sites that were selected from each administrative zone, the total number of attendees who were screened for pulmonary TB (PTB) symptoms, the number of attendees who had PTB suggestive symptoms, the number of bacteriologically confirmed cases, and the number of individuals who were Löwenstein–Jensen culture-negative test result.

**Table S2** shows the proportion of PTB positivity by gender and age group. The proportion estimation analysis revealed that the prevalence of culture-positive PTB was nearly equal for males and females (21.8%±2.35SE Vs 21.8%±2.6SE). Also, participants aged 18 to 33 years had a higher proportion of culture-positive PTB (28.5% ± 2.8SE).

**Table S3** summarizes the bivariate logistic regression analysis of socio-demographic characteristics of participants and associated risk factors for culture-positive PTB. The analysis revealed that participants aged 34-49 years and rural residents were statistically associated with culture-positive PTB (*p* <0.05). Furthermore, the analysis revealed that few independent variables were statistically associated with culture-positive PTB (*p* ≤ 0.01), including a history of TB disease, contact with chronic coughers or active TB patients, having had close contact with a family member who has TB, the number of days spent (>21) at HWS, and sharing living spaces at HWS.

In **Table S4**, we compute the proportion of any drug-resistant TB and MDR-TB among each age group of participants in each study area. This allows us to assess the extent to which each age group appears to be affected by any drug-resistant TB and MDR-TB strains. Similarly, as illustrated in **Table S5,** we used a logistic regression analysis model to determine the odds of developing any drug-resistant TB and MDR-TB among participants in each study site.

**Tables S6 & S7** Provide detailed data about participants who had culture-negative test results whilst at HWS. **Table S6** illustrates the profiles of participants and the proportion of developing active TB disease post-exposure to HWS among those who had culture-negative results whilst at HWS. Besides, **Table S7** shows the bivariate and multivariate logistic regression analysis of the sociodemographic characteristics of participants and associated factors among those who reported contracted active TB disease post-exposure to HWS.

| Table S1: Geographical location of the study area (administrative zones), spiritual HWSs, the number of individuals screened for pulmonary TB suggestive symptoms, enrolled participants in each study site, and LJ culture results, 2019-2020. | | | | | | | |
| --- | --- | --- | --- | --- | --- | --- | --- |
| S. No | **Study area (zones)** | **Holy Water Site (HWS)** | **No. of attendees screened for PTB symptoms** | **No. of anticipated PTB symptomatic attendees to be recruited** | **No. of participants (individuals with PTB symptoms),** | **Bacteriologically confirmed PTB cases** | **Culture negative case** |
| 1 | North-Wello | *Urael* | 1458 | 75 | 107 | 22 | 85 |
| 2 | South-Wello | *Amanual* | 1601 | 75 | 100 | 22 | 78 |
| 3 | North-Shewa | *Tsadkane Mariam* | 3221 | 75 | 97 | 33 | 64 |
| 4 | South-Gondar | *Fogera -Arsema* | 1201 | 75 | 105 | 28 | 77 |
| 5 | Central-Gondar | *Teklehymanot* | 820 | 75 | 30 | 2 | 28 |
| 6 | Awi zone | *Ashewa Medihanealem* | 530 | 75 | 22 | 2 | 20 |
| 7 | West-Gojam | *Zera-biruk* | 471 | 75 | 38 | 4 | 34 |
| 8 | Wag-Hamra | *Gihorgis* | 543 | 75 | 36 | 5 | 31 |
| 9 | East-Gojam | *Washa-Giyorgis* | 468 | 75 | 25 | 4 | 21 |
|  | **Total** | | **10313** | **675** | **560** | **122** | **438** |
| *Note:PTB: Pulmonary tuberculosis; LJ: Lowenstein-Jensen; HWS: Holy water sites.* | | | | | | | |

**Table S2:** The proportion of participants with culture-positive PTB by gender and age category, (number of observations = 560).

|  | Proportion | Std. Err. | Logit  [95% Conf. Interval] | |
| --- | --- | --- | --- | --- |
| PTB positivity status by gender |  |  |  |  |
| Negative: Male | 0.782 | 0.024 | 0.733 | 0.825 |
| Negative: Female | 0.782 | 0.026 | 0.726 | 0.829 |
| Positive: Male | 0.218 | 0.024 | 0.175 | 0.267 |
| Positive: Female | 0.218 | 0.026 | 0.171 | 0.274 |
|  |  |  |  |  |
| PTB positivity status by age group (year) |  |  |  |  |
| Negative: 18 - 33 | 0.715 | 0.028 | 0.657 | 0.766 |
| Negative: 34 - 49 | 0.851 | 0.025 | 0.796 | 0.893 |
| Negative: ≥ 50 | 0.820 | 0.041 | 0.726 | 0.887 |
| Positive: 18 - 33 | 0.285 | 0.028 | 0.234 | 0.343 |
| Positive: 34 - 49 | 0.149 | 0.025 | 0.107 | 0.204 |
| Positive: ≥50 | 0.180 | 0.041 | 0.113 | 0.274 |

**Table S3:** Bivariate logistic regression analysis of socio-demographic characteristics of participants and potential risk factors for culture-positive TB (n = 560).

| Variables | | Prevalence of culture-positive PTB | | | cOR (95%CI) | *p*-value |
| --- | --- | --- | --- | --- | --- | --- |
|  |  | **Positive (n)** | **Negative (n)** |  | |  |
| Sex | Male | 67 | 241 | 0.995 [0.67-1.50] | | 0.984 |
|  | Female | 55 | 197 | ref | |  |
| Age group (year) | 18-33 | 75 | 188 | ref | |  |
|  | 34-49 | 31 | 177 | 0.44 [0.28-0.70] | | 0.001 |
|  | ≥ 50 | 16 | 73 | 0.55 [0.30-1.00] | | 0.052 |
| Residence | Urban | 46 | 212 | ref | |  |
|  | Rural | 76 | 226 | 1.55 [1.03-2.34] | | 0.037 |
| Marital status | Married | 85 | 271 | 1.42 [0.92-2.18] | | 0.114 |
|  | Single* | 37 | 167 | ref | |  |
| Educational status | Can't read and write | 59 | 197 | 0.96 [0.59-1.57] | | 0.876 |
|  | Primary school | 30 | 135 | 0.71 [0.41-1.24] | | 0.235 |
|  | Secondary school & above | 33 | 106 | ref | |  |
| Household size | 1-5 | 57 | 237 | ref | |  |
|  | > 5 | 65 | 201 | 1.34 [0.89-2.01] | | 0.149 |
| Occupation | Farmer | 45 | 190 | ref | |  |
|  | Employed **^a^** | 6 | 18 | 1.41 [0.53-3.75] | | 0.494 |
|  | Unemployed ^b^ | 23 | 94 | 1.03 [0.59-1.81] | | 0.909 |
|  | Housewife | 24 | 70 | 1.45 [0.82-2.54] | | 0.200 |
|  | Students & others** | 24 | 66 | 1.54 [0.87-2.71] | | 0.140 |
|  | | | | | | |
| Heard about PTB disease | Yes | 93 | 306 | ref | |  |
|  | No | 29 | 132 | 0.72 [0.45-1.15] | | 0.171 |
| Knowing PTB is a transmittable disease | Yes | 34 | 156 | ref | |  |
|  | No | 88 | 282 | 1.43 [0.92-2.22] | | 0.11 |
| Had PTB disease before | Yes | 41 | 70 | 2.66 [1.68-4.19] | | < 0.001 |
|  | No | 81 | 368 | ref | |  |
| History of contact with chronic coughers | Yes | 78 | 119 | 4.75 [3.11-7.27] | | < 0.001 |
|  | No | 44 | 319 | ref | |  |
| History of contact with TB patients | Yes | 78 | 113 | 5.10 [3.32-7.81] | | < 0.001 |
|  | No | 44 | 325 | ref | |  |
| Has anyone in your family or other close contact had TB? | Yes | 77 | 109 | 5.16 [3.37-7.91] | | < 0.001 |
|  | No | 45 | 329 | ref | |  |
| History of the previous stay at the HWS (in the past year). | Yes | 105 | 240 | 5.10 [2.95-8.80] | | < 0.001 |
|  | No | 17 | 198 | ref | |  |
| Number of days spent at the HWS | ≤ 21 days | 25 | 205 | ref | |  |
|  | >21 days | 97 | 233 | 3.41 [2.12-5.51] | | < 0.001 |
| Sharing drinking cups at the HWS | Yes | 91 | 268 | 0.54 [0.34-0.84] | | 0.01 |
|  | No | 31 | 170 | ref | |  |
| Sharing a living room at the HWS | Yes | 118 | 320 | 10.88 [3.92-30.12] | | < 0.001 |
|  | No | 4 | 118 | ref | |  |
| Ever being on TB medication | Yes | 42 | 66 | 2.96 [1.88-4.67] | | < 0.001 |
|  | No | 80 | 372 | ref | |  |
| Did you complete the medication? (n=108) | Yes | 29 | 50 | ref | |  |
|  | No | 13 | 16 | 1.40 [1.19-2.92] | | 0.44 |
| What was the year of the last episode of your treatment? | Post-2006 E.C | 24 | 37 | ref | |  |
|  | ≤ 2006 E.C | 18 | 29 | 0.96 [0.43-2.09] | | 0.91 |
|  | NA | 80 | 372 | 0.33 [0.19-0.58] | | < 0.001 |
| Where did you take the TB treatment? (n=108) | Hospital | 14 | 22 | 6.10 [0.14-45.20] | | 1.00 |
|  | Health Center | 27 | 36 | 6.00 [0.71-50.90] | | 1.40 |
|  | Private clinic | 1 | 8 | ref | |  |
| Habits of cigarette smoking | Yes | 8 | 37 | 0.76 [0.34-1.68] | | 0.49 |
|  | No | 114 | 401 | ref | |  |
| How long did you smoke a cigarette? (n=45) | ≤ 5 years | 4 | 28 | ref | |  |
|  | >5 years | 4 | 9 | 3.10 [0.64-15.05] | | 0.16 |
| Habits of alcohol drinking | Yes | 5 | 39 | 0.44 [0.17-1.13] | | 0.09 |
|  | No | 117 | 399 | ref | |  |
| Do you know your HIV status? | Yes | 34 | 179 | ref | |  |
|  | No | 88 | 259 | 1.79 [1.15-2.78] | | 0.01 |
| Do you have diabetes mellitus? | Yes | 3 | 30 | 0.47 [0.14-1.60] | | 0.22 |
|  | No | 47 | 220 | ref | |  |
|  | Don't know | 72 | 188 | 1.79 [1.18-2.72] | | 0.01 |
| Do you have hypertension? | Yes | 4 | 33 | 0.54 [0.18-1.60] | | 0.27 |
|  | No | 50 | 224 | ref | |  |
|  | Don't know | 68 | 181 | 1.68 [1.11-2.55] | | 0.014 |
| Have you received any cancer treatment recently? | Yes | 3 | 20 | 1.90 [0.55-6.50] | | 0.31 |
|  | No | 119 | 418 | ref | |  |
| Have you been admitted to the hospital this year? | Yes | 15 | 65 | 1.24 [0.68-2.27] | | 0.71 |
|  | No | 107 | 373 | ref | |  |
| *Notes: * Single, divorced & widowed; **religious leaders & deacon; ref: reference. Abbreviations: CI: confidence interval; cOR: crude odds ratio; E.C: Ethiopian calendar; HIV: Human immunodeficiency virus; HWS: Holy water site/s; PTB: pulmonary tuberculosis; ref: reference; TB: Tuberculosis; ^a^ construction worker, administrative worker, healthcare worker, public transport worker; ^b^ businessman, trader, daily laborer.* | | | | | | |

**Table S****4:** The proportion of any drug-resistant TB and MDR-TB among each age group of participants in each study area (zone).

| The proportion of any drug-resistant TB (number of observations = 20). | | | | | | |
| --- | --- | --- | --- | --- | --- | --- |
| Study area by Age group (year) | | **Proportion** | **Std. Err.** | **Logit**  **[95% Conf. Interval]** | |  |
| North Wello: | 18 - 33 | 0.059 | 0.057 | 0.007 | 0.351 |  |
| North Wello: | ≥ 50 | 0 | (No of observations) | - | - |  |
| South Wello: | 18 - 33 | 0.471 | 0.121 | 0.243 | 0.711 |  |
| South Wello: | ≥ 50 | 0.333 | 0.272 | 0.037 | 0.866 |  |
| North Shewa: | 18 - 33 | 0.118 | 0.078 | 0.027 | 0.392 |  |
| North Shewa: | ≥ 50 | 0.333 | 0.272 | 0.037 | 0.866 |  |
| South Gondar: | 18 - 33 | 0.294 | 0.111 | 0.120 | 0.559 |  |
| South Gondar: | ≥ 50 | 0.333 | 0.272 | 0.037 | 0.866 |  |
| C.Gondar & others*: | 18 - 33 | 0.059 | 0.057 | 0.007 | 0.351 |  |
| C. Gondar & others*: | ≥ 50 | 0 | (no of obs) | - | - |  |
| The proportion of MDR-TB (resistance to both RIF and INH) (number of observations =15). | | | | | | |
| Study area | **Age (year)** | **Proportion** | **Std. Err.** | **Logit**  **[95% Conf. Interval]** | |  |
| North Wello: | 18 - 33 | 0.083 | 0.080 | 0.010 | 0.461 |  |
| North Wello: | ≥ 50 | 0 | (No of obs) | - | - |  |
| South Wello: | 18 - 33 | 0.583 | 0.142 | 0.285 | 0.831 |  |
| South Wello: | ≥ 50 | 0.333 | 0.272 | 0.035 | 0.874 |  |
| North Shewa: | 18 - 33 | 0.167 | 0.108 | 0.037 | 0.513 |  |
| North Shewa: | ≥ 50 | 0.333 | 0.272 | 0.035 | 0.874 |  |
| South Gondar: | 18 - 33 | 0.083 | 0.080 | 0.010 | 0.461 |  |
| South Gondar: | ≥ 50 | 0.333 | 0.272 | 0.035 | 0.874 |  |
| C.Gondar & other*: | 18 - 33 | 0.083 | 0.080 | 0.010 | 0.874 |  |
| C.Gondar & other*: | ≥ 50 | 0 | (no obs) | - | - |  |
| *Notes: *Others: Awi, West gojjam, Wag-Hamra, East gojjam zone. Abbreviations: INH: isoniazid; MDR: multidrug resistant; RIF: rifampicin; TB: tuberculosis.* | | | | | | |

**Table S5:** Logistic regression analysis of the occurrence of any drug-resistant TB (RIF^r^ and/or INH^r^) and MDR-TB in each study area (number of observations = 122).

| The occurrence of any drug-resistant TB (RIF^r^ and/or INH^r^) | | | | | | | |
| --- | --- | --- | --- | --- | --- | --- | --- |
| Logistic Regression    Log Likelihood = -47.355968 | | | | Number of obs = 122  LR Chi^2^ (4) = 14.15  Prob > Chi^2^ = 0.0068  Pseudo R2 = 0.1299 | | | |
| The study area (zones) | **Odds Ratio** | **Std. Err.** | **z** | **P>\|z\|** | **[95% Conf. Interval]** | | |
| North Wello | 1 | (base) | **-** | **-** | **-** | **-** | |
| South Wello | 14.54 | 16.16 | 2.41 | 0.016 | 1.65 | 128.45 | |
| North Shewa | 2.1 | 2.50 | 0.62 | 0.533 | 0.20 | 21.60 | |
| South Gondar | 5.73 | 6.43 | 1.55 | 0.120 | 0.63 | 51.68 | |
| Central Gondar & Others* | 1.31 | 1.91 | 0.19 | 0.852 | 0.08 | 22.62 | |
| _Cons | 0.05 | 0.05 | -2.97 | 0.003 | 0.01 | 0.35 | |
| The occurrence of MDR-TB (resistance to both RIF and INH) | | | | | | | |
| Study area (zones) | **Odds ratio** | **Std. Err.** | **z** | **P>\|z\|** | **[95% Conf. Interval]** | | |
| North Wello | 1 | (base) | **-** | **-** | **-** | **-** | |
| South Wello | 12.00 | 13.38 | 2.23 | 0.026 | 1.35 | 106.80 | |
| North Shewa | 2.10 | 2.50 | 0.62 | 0.533 | 0.20 | 21.60 | |
| South Gondar | 1.62 | 2.03 | 0.38 | 0.703 | 0.14 | 19.07 | |
| C.Gondar & Others* | 1.31 | 1.91 | 0.19 | 0.852 | 0.08 | 22.62 | |
| _Cons | 0.05 | 0.05 | -2.97 | 0.003 | 0.01 | 0.35 | |
| *Note: _Cons Estimates baseline odds; *Others: Awi, West gojjam, Wag-Hamra, East gojjam. Abbreviations: INH: isoniazid; INH^r^: isoniazid-resistant; MDR: Multidrug-resistant; RIF: rifampicin; RIF^r^: rifampicin-resistant; TB: tuberculosis.* | | | | | | | |
|  | | | | | | |  |

**Table S6**: Proportion of participants with culture-negative test results whilst at HWS, and reported the development of active TB post-exposure to HWS (n = 438).

| Socio-demographic characteristics | | Total | Develop active TB disease post-exposure to HWS | | *p*-value |
| --- | --- | --- | --- | --- | --- |
|  |  |  | **Yes, n (%)** | **No, n (%)** |  |
| Sex | Male | 241 | 23 (9.5) | 218 (90.5) | 0.014 |
|  | Female | 197 | 7 (3.6) | 190 (96.4) |  |
| Age group (year) | 18-33 | 188 | 4 (2.1) | 184 (97.9) | < 0.001 |
|  | 34-49 | 177 | 12 (6.8) | 165 (93.2) |  |
|  | ≥ 50 | 73 | 14 (19.2) | 59 (80.8) |  |
| Residence | Urban | 212 | 7 (3.3) | 205 (96.7) | 0.004 |
|  | Rural | 226 | 23 (10.2) | 203 (89.8) |  |
| Marital status | Married | 271 | 23 (8.5) | 248 (91.5) | 0.118 |
|  | Single* | 167 | 7 (4.2) | 160 (95.8) |  |
| Educational status | Can’t read and write | 197 | 25 (12.7) | 172 (87.3) | < 0.001 |
|  | Primary school & above | 241 | 5 (2.1) | 236 (97.9) |  |
| Household size | 1-5 | 237 | 8 (3.4) | 229 (96.6) | 0.002 |
|  | > 5 | 201 | 22 (10.9) | 179 (89.1) |  |
| Occupation | Farmer | 190 | 21 (11.1) | 169 (88.9) | 0.012 |
|  | Unemployed | 112 | 4 (3.6) | 108 (96.4) |  |
|  | Housewife & others** | 136 | 5 (3.7) | 131 (96.3) |  |
| *NB: * Single, divorced & widowed; ** Student; religious leaders & deacon; HWS: Holy water sites; TB: tuberculosis.* | | | | | |

**Table S7:** Bivariate and multivariate logistic regression analysis of socio-demographic characteristics of participants and risk factors for developing active TB diseases among those who reported developing active TB disease post-residency at the HWSs [(Number of participants who were culture (-) whilst at HWS (n = 438)].

| Variables  (Socio-demographic profiles and risk factors) | | | Develop active TB post-exposure to HWS | | | | cOR (95%CI) | | *p-*value | *aOR (95%CI)* | *p-*value | |  |  |
| --- | --- | --- | --- | --- | --- | --- | --- | --- | --- | --- | --- | --- | --- | --- |
|  |  |  | **Yes, (n)** | | **No, (n)** | |  |  |  |  |  |  |  |  |
| Sex | Male | 23 | | 218 | | ref | |  | | ref | |  | |  |
|  | Female | 7 | | 190 | | 2.86 [1.20-14.57] | | 0.018 | | 8.43 [1.90-36.70] | | 0.004 | |  |
| Age group (year) | 18-33 | 4 | | 184 | | ref | |  | | ref | |  | |  |
|  | 34-49 | 12 | | 165 | | 3.35 [1.06-10.58] | | 0.04 | | 2.38 [0.44-12.73] | | 0.311 | |  |
|  | ≥50 | 14 | | 59 | | 10.92 [3.46-34.45] | | ≤ 0.001 | | 5.98 [0.92-35.06] | | 0.062 | |  |
| Residence | Urban | 7 | | 205 | | ref | |  | | ref | |  | |  |
|  | Rural | 23 | | 203 | | 3.32 [1.39-7.90] | | 0.007 | | 0.818 [0.13-5.00] | | 0.82 | |  |
| Marital status | Married | 23 | | 248 | | 2.12 [0.89-5.06] | | 0.09 | | 0.875 [0.28-2.76] | | 0.82 | |  |
|  | Single* | 7 | | 160 | | ref | |  | | ref | |  | |  |
| Educational status | None | 25 | | 172 | | 6.86 [2.74-18.28] | | ≤ 0.001 | | 6.09 [1.25-29.55] | | 0.025 | |  |
|  | Primary school & above | 5 | | 236 | | ref | |  | | ref | |  | |  |
| Household size | 1-5 | 8 | | 229 | | ref | |  | | ref | |  | |  |
|  | > 5 | 22 | | 179 | | 3.52 [1.53-8.09] | | 0.003 | | 3.31 [0.98-11.16] | | 0.053 | |  |
|  |  |  | |  | |  | |  | |  | |  | |  |
| Heard about TB disease | Yes | 21 | | 285 | | ref | | 0.99 | | ref | |  | |  |
|  | No | 9 | | 123 | | 0.99 [0.44-2.23] | |  | | 0.65 [0.19-2.18] | | 0.48 | |  |
| Know TB is a transmittable disease | Yes | 6 | | 150 | | ref | | 0.07 | | ref | |  | |  |
|  | No | 24 | | 258 | | 2.33 [0.93-5.82] | |  | | 2.93 [0.67-12.81] | | 0.15 | |  |
| Had TB disease before? | Yes | 3 | | 67 | | ref | | 0.36 | | ref | |  | |  |
|  | No | 27 | | 341 | | 1.77 [0.52-6.00] | |  | | 0.78 [0.04-17.14] | | 0.88 | |  |
| History of contact with chronic coughers | Yes | 13 | | 106 | | 2.18 [1.02-4.60] | | 0.04 | | 0.52 [0.04- 6.34] | | 0.69 | |  |
|  | No | 17 | | 302 | | ref | |  | | ref | |  | |  |
| History of contact with TB patients | Yes | 11 | | 102 | | 1.73 [0.80-3.77] | | 0.16 | | 0.12 [0.01-2.19] | | 0.15 | |  |
|  | No | 19 | | 306 | | ref | |  | | ref | |  | |  |
| Has anyone in your family or other close contact had TB? | Yes | 14 | | 95 | | 2.88 [1.36-6.12] | | 0.006 | | 25.20 [3.01-206.50] | | 0.003 | |  |
|  | No | 16 | | 313 | | ref | |  | | ref | |  | |  |
| History of the previous stay at HWS | Yes | 24 | | 216 | | 3.56 [1.42-8.88] | | 0.007 | | - | | - | |  |
|  | No | 6 | | 192 | | ref | |  | | - | | - | |  |
| Number of days spent at HWS | ≤ 21 days | 8 | | 197 | | ref | | 0.026 | | - | | - | |  |
|  | >21 days | 22 | | 211 | | 2.57 [1.12-5.90] | |  | | - | | - | |  |
| Sharing drink cups at HWS | Yes | 24 | | 244 | | 0.37 [0.15-0.93] | | 0.034 | | 0.03 [0.06-0.99] | | 0.05 | |  |
|  | No | 6 | | 164 | | ref | |  | | ref | |  | |  |
| Sharing living space at the HWS | Yes | 29 | | 291 | | 11.66 [1.57-86.59] | | 0.016 | | 37.19 [2.46-561.2] | | 0.009 | |  |
|  | No | 1 | | 117 | | ref | |  | | ref | |  | |  |
| Ever being on TB medication | Yes | 3 | | 63 | | 0.61[0.18-2.07] | | 0.42 | | 8.81 [0.17-450.5] | | 0.28 | |  |
|  | No | 27 | | 345 | | ref | |  | | ref | |  | |  |
| Did you complete the medication?  What was the year of the last episode of your treatment? | Yes | 2 | | 48 | | ref | | 0.71 | | ref | |  | |  |
|  | No | 1 | | 15 | | 1.60 [0.14-18.9] | |  | | 6.75 [0.23-191.0] | | 0.26 | |  |
|  | Post-2006 E.C | 2 | | 35 | | ref | | 0.71 | | ref | |  | |  |
|  | ≤2006 E.C | 1 | | 28 | | 0.63 [0.05-7.25] | |  | | 0.173 [0.01-3.44] | | 0.250 | |  |
| Habits of cigarette smoking | Yes | 1 | | 36 | | 0.36 [0.05-2.69] | | 0.32 | | - | | - | |  |
|  | No | 29 | | 372 | | ref | |  | | - | |  | |  |
| Habits of alcohol drinking | Yes | 3 | | 36 | | 1.14 [0.33-3.97] | | 0.22 | | - | | - | |  |
|  | No | 27 | | 372 | | ref | |  | | - | |  | |  |
| Habits of alcohol drinking | Yes | 3 | | 36 | | 1.14 [0.33-3.97] | | 0.22 | | - | | - | |  |
|  | No | 27 | | 372 | | ref | |  | | - | |  | |  |
| Do you know your HIV status? | Yes | 13 | | 166 | | ref | | 0.78 | | ref | |  | |  |
|  | No | 17 | | 242 | | 0.90 [0.42-1.90] | |  | | 0.35 [0.10-1.30] | | 0.119 | |  |
| Do you have diabetes mellitus? | Yes | 2 | | 28 | | 0.80 [0.18-3.64] | | 0.78 | | 0.314 [0.03-3.63] | | 0.350 | |  |
|  | No | 18 | | 202 | | ref | |  | | ref | |  | |  |
| Do you have hypertension? | Yes | 4 | | 29 | | 1.73 [0.56-5.73] | | 0.33 | | 3.97 [0.48-6.11] | | 0.20 | |  |
|  | No | 16 | | 208 | | ref | |  | | ref | |  | |  |
| Have you received any cancer treatment recently? | Yes | 1 | | 19 | | 1.42 [0.18-10.96] | | 0.74 | | 3.35 [0.180-61.0] | | 0.42 | |  |
|  | No | 29 | | 389 | | ref | |  | | ref | |  | |  |
| Have you been admitted to the hospital in the last 1 year? | Yes | 2 | | 63 | | 2.56 [0.29-11.0] | | 0.21 | | 14.5 [1.16-180.9] | | 0.04 | |  |
|  | No | 28 | | 345 | | ref | |  | | ref | |  | |  |
| *Note: *Single, divorced & widowed; ^**^ Student, religious leaders & deacons; Abbreviations: aOR: adjusted odds ratio; CI: confidence interval;*  *cOR: crude odds ratio; E.C: Ethiopian calendar; HIV: Human immunodeficiency virus; HWS: holy water site; PTB: Pulmonary tuberculosis; ref: reference; TB: Tuberculosis.* | | | | | | | | | | | | | | |
